# Supplementary material for: Conditional chemoconnectomics (cCCTomics) as a strategy for efficient and conditional targeting of chemical transmission
Source: eLife. 2024 Apr 30;12:RP91927. doi: 10.7554/eLife.91927 (PMC11060718; doi:10.7554/eLife.91927)
Supplement: Figure 3—source data 3. [file elife-91927-fig3-data3.zip › Figure 3 source data3 part 2.zip!/Figure 3-Source data 3-part2/1002-9-CRISPResso_Report_e53dd8m5.zip!/CRISPResso_on_1002_10/3b.Insertion_deletion_substitutions_size_hist.pdf]

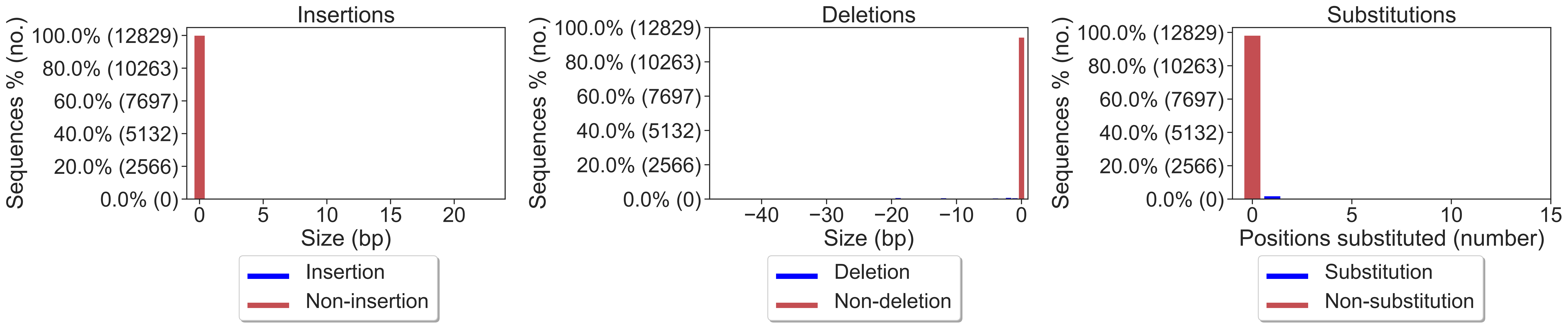
Insertions
Sequences % (no.)
100.0% (12829)
80.0% (10263)
60.0% (7697)
40.0% (5132)
20.0% (2566)
0.0% (0)
0
5
10
15
20
Size (bp)
Insertion
Non-insertion
Deletions
−40
−30
−20
−10
0
Deletion
Non-deletion
Substitutions
Positions substituted (number)
Substitution
Non-substitution
